# Acetonitrilebis(2,9-dimethylphenanthroline)copper(II) bis(tetrafluoridoborate) acetonitrile disolvate

**DOI:** 10.1107/S1600536809012331

**Published:** 2009-04-30

**Authors:** Stephen P. Watton

**Affiliations:** a Department of Chemistry, Central Connecticut State University, 1615 Stanley Street, New Britain, CT 06050, USA

## Abstract

In the title compound, [Cu(CH_3_CN)(C_14_H_12_N_2_)_2_](BF_4_)_2_·2CH_3_CN, the Cu^II^ atom shows a distorted CuN_5_ square-pyramidal geometry with the acetonitrile N atom in an equatorial site, which differs substanti­ally from the distorted trigonal-bipyramidal arrangement usually observed for five-coordinate complexes of Cu^II^ with two phenanthroline-type ligands and one other ligand. The B atom of one of the BF_4_^−^ anions is disordered over two sites in a 0.825 (2):0.175 (2) ratio. In the crystal, C—H⋯F hydrogen bonds help to establish the packing.

## Related literature

For related structures, see: Bush *et al.* (2001 ▶); Vega *et al.* (1985 ▶); Aligo *et al.* (2005 ▶). For background, see: Kepert (1973 ▶); Rossi & Hoffman (1975 ▶); James & Williams (1961 ▶).
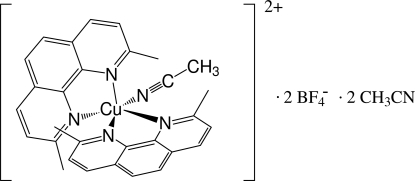


## Experimental

### 

#### Crystal data

[Cu(C_2_H_3_N)(C_14_H_12_N_2_)_2_](BF_4_)_2_·2C_2_H_3_N
*M* *_r_* = 776.83
Triclinic, 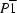

*a* = 11.2865 (19) Å
*b* = 12.070 (2) Å
*c* = 13.802 (2) Å
α = 72.843 (15)°
β = 83.746 (15)°
γ = 73.933 (15)°
*V* = 1725.7 (5) Å^3^
*Z* = 2
Mo *K*α radiation
μ = 0.71 mm^−1^
*T* = 293 K
0.3 × 0.2 × 0.2 mm

#### Data collection

Oxford Diffraction Sapphire diffractometer
Absorption correction: multi-scan (*SCALE3 ABSPACK* in *CrysAlis RED*; Oxford Diffraction, 2006 ▶). *T*_min_ = 0.997, *T*_max_ = 1.000 (expected range = 0.864–0.867)
10543 measured reflections
8129 independent reflections
4935 reflections with *I* > 2σ(*I*)
*R*_int_ = 0.025

#### Refinement

*R*[*F* ^2^ > 2σ(*F* ^2^)] = 0.040
*wR*(*F* ^2^) = 0.098
*S* = 0.89
8129 reflections
489 parameters
30 restraints
H-atom parameters constrained
Δρ_max_ = 0.54 e Å^−3^
Δρ_min_ = −0.35 e Å^−3^

### 

Data collection: *CrysAlis CCD* (Oxford Diffraction, 2006 ▶); cell refinement: *CrysAlis RED* (Oxford Diffraction, 2006 ▶); data reduction: *CrysAlis RED*; program(s) used to solve structure: *SHELXS97* (Sheldrick, 2008 ▶); program(s) used to refine structure: *SHELXL97* (Sheldrick, 2008 ▶); molecular graphics: *ORTEP-3* (Farrugia, 1997 ▶); software used to prepare material for publication: *SHELXTL* (Sheldrick, 2008 ▶).

## Supplementary Material

Crystal structure: contains datablocks global, I. DOI: 10.1107/S1600536809012331/hb2926sup1.cif

Structure factors: contains datablocks I. DOI: 10.1107/S1600536809012331/hb2926Isup2.hkl

Additional supplementary materials:  crystallographic information; 3D view; checkCIF report

## Figures and Tables

**Table:** 

Cu1—N5	2.0123 (18)
Cu1—N2	2.0297 (17)
Cu1—N3	2.0305 (18)
Cu1—N4	2.0348 (17)
Cu1—N1	2.1760 (18)
N3—C25	1.351 (3)
N3—C28	1.359 (3)
N4—C16	1.333 (2)
N4—C27	1.365 (3)

**Table:** 

N5—Cu1—N2	84.42 (7)
N5—Cu1—N3	89.07 (7)
N2—Cu1—N3	165.23 (7)
N5—Cu1—N4	150.90 (7)
N2—Cu1—N4	98.14 (7)
N3—Cu1—N4	81.25 (7)
N5—Cu1—N1	100.93 (7)
N2—Cu1—N1	80.17 (7)
N3—Cu1—N1	114.15 (7)
N4—Cu1—N1	108.09 (6)

**Table 2:** Hydrogen-bond geometry (Å, °)

*D*—H⋯*A*	*D*—H	H⋯*A*	*D*⋯*A*	*D*—H⋯*A*
C12—H12*C*⋯F8^i^	0.96	2.36	3.120 (3)	135
C18—H18⋯F5^ii^	0.93	2.36	3.279 (3)	171
C20—H20⋯F8^ii^	0.93	2.53	3.423 (3)	161
C30—H30*A*⋯F8	0.96	2.47	3.375 (4)	158
C30—H30*B*⋯F6^iii^	0.96	2.38	3.314 (3)	165
C32—H32*B*⋯F7^iv^	0.96	2.37	3.191 (3)	143

Symmetry codes: (i) 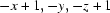; (ii) 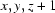; (iii) 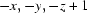; (iv) 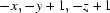.

**Fig. 1.**
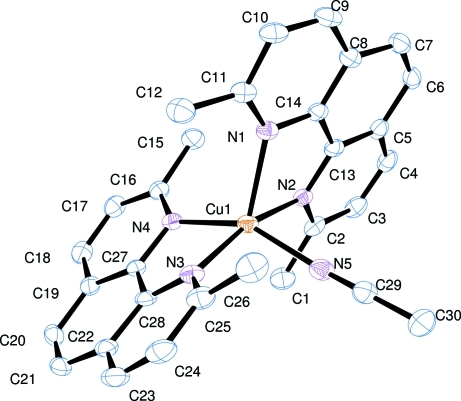
View of the cation in (I) with H atoms omitted for clarity. Displacement ellipsoids are drawn at the 50% probability level.

**Fig. 2.**
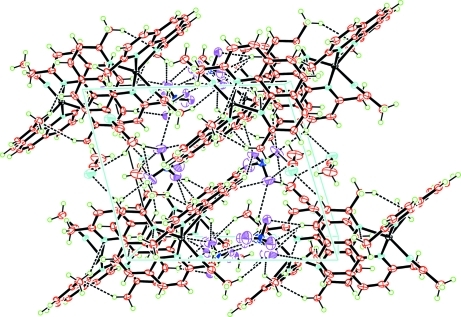
The packing for (I): H atoms are omitted for clarity and displacement ellipsoids are drawn at the 50% probability level.

## supplementary crystallographic information

### Comment

The copper-containing cation (Fig. 1) of the title compound, (I), consists of a 5-coordinate Cu center, with the geometry about copper being best described (Kepert, 1973) as distorted square pyramidal, rather than as a distorted TBP structure, which is most commonly observed for five-coordinate copper bis-phenanthroline complexes (Bush, *et al.*, 2001).  Most of the distortion from idealized square pyramidal can be explained in terms of the restricted bite angles of the rigid phenanthroline rings. It is assumed that steric strain associated with the presence of the 2,9-dimethyl groups on the ligand overrides electronic considerations (Rossi and Hoffman, 1975), resulting in formation of the disfavored square pyramidal geometry for the d^9^ complex.  The steric strain inherent in the structure is also reflected in the copper being located considerably outside of the normal coordination plane of the phenanthroline [0.470 (1) and 0.636 (1)Å from the least squares planes of the two rings], and in a clear bowing of the phenanthroline ligand itself.

The observation of an electronically high-energy structure is fully consistent with electrochemical data (James and Williams, 1961) which show the 2,9-disubstituted phenanthroline complex to be significantly easier to reduce than the analogous complexes lacking the 2,9- substituents. Reduction of the [Cu(neocuproine)_2_(solv)]^2+^ complexes affords the air-stable [Cu(neocuproine)_2_]^+^ species, which adopt pseudo-tetrahedral geometries that alleviate the steric strain between the substituents.

In the structure of (I), close contact of the disordered BF_4_^-^ and the coordinated CH_3_CN suggest that a C—H hydrogen bonding interaction exists (see, for example: Vega, *et al.*, 1985). Similar interactions between BF_4_^-^ ions and Cu-bound acetonitrile ligands have been observed previously (Aligo, *et al.*, 2005). The packing of (I) is shown in Fig. 2 and the H bonds are listed in Table 2.

### Experimental

Copper (II) tetrafluoroborate hydrate (0.100 g, 0.42 mmol) was dissolved in 5 ml of acetonitrile and 2,9-dimethylphenanthroline (0.190 g, 0.92 mmol) was added as a solution in 5 ml of acetonitrile. Vapor diffusion of ether into the solution afforded green blocks of (I). Yield 0.182 g (56%).

### Refinement

All H atoms were included at calculated positions and were allowed to ride with their C atoms during refinement. The structure exhibits disorder of one of the two BF_4_^-^ counterions. The disorder was modeled using two identical constrained fragments which shared a common F atom. Occupancy refinement indicated a relative population of 0.175 (2) to 0.825 (2) for the two positions.

### Crystal data

**Table:**

[Cu(C_2_H_3_N)(C_14_H_12_N_2_)_2_](BF_4_)_2_·2C_2_H_3_N	*Z* = 2
*M**_r_* = 776.83	*F*(000) = 794
Triclinic, *P*1	*D*_x_ = 1.495 Mg m^−^^3^
Hall symbol: -P 1	Melting point > 523 K
*a* = 11.2865 (19) Å	Mo *K*α radiation, λ = 0.71073 Å
*b* = 12.070 (2) Å	Cell parameters from 4074 reflections
*c* = 13.802 (2) Å	θ = 3.9–32.1°
α = 72.843 (15)°	µ = 0.71 mm^−^^1^
β = 83.746 (15)°	*T* = 293 K
γ = 73.933 (15)°	Block, green
*V* = 1725.7 (5) Å^3^	0.3 × 0.2 × 0.2 mm

### Data collection

**Table:**

Oxford Diffraction Sapphire diffractometer	8129 independent reflections
Radiation source: fine-focus sealed tube	4935 reflections with *I* > 2σ(*I*)
graphite	*R*_int_ = 0.025
ω scans	θ_max_ = 32.2°, θ_min_ = 3.9°
Absorption correction: multi-scan (SCALE3 ABSPACK in *CrysAlis RED*; Oxford Diffraction, 2006).	*h* = −16→13
*T*_min_ = 0.997, *T*_max_ = 1.000	*k* = −17→17
10543 measured reflections	*l* = −20→18

### Refinement

**Table:**

Refinement on *F*^2^	Primary atom site location: structure-invariant direct methods
Least-squares matrix: full	Secondary atom site location: difference Fourier map
*R*[*F*^2^ > 2σ(*F*^2^)] = 0.040	Hydrogen site location: inferred from neighbouring sites
*wR*(*F*^2^) = 0.098	H-atom parameters constrained
*S* = 0.89	*w* = 1/[σ^2^(*F*_o_^2^) + (0.0558*P*)^2^] where *P* = (*F*_o_^2^ + 2*F*_c_^2^)/3
8129 reflections	(Δ/σ)_max_ = 0.009
489 parameters	Δρ_max_ = 0.54 e Å^−^^3^
30 restraints	Δρ_min_ = −0.35 e Å^−^^3^

### Special details

**Table:**

Geometry. All e.s.d.'s (except the e.s.d. in the dihedral angle between two l.s. planes) are estimated using the full covariance matrix. The cell e.s.d.'s are taken into account individually in the estimation of e.s.d.'s in distances, angles and torsion angles; correlations between e.s.d.'s in cell parameters are only used when they are defined by crystal symmetry. An approximate (isotropic) treatment of cell e.s.d.'s is used for estimating e.s.d.'s involving l.s. planes.
Refinement. Refinement of *F*^2^ against ALL reflections. The weighted *R*-factor *wR* and goodness of fit *S* are based on *F*^2^, conventional *R*-factors *R* are based on *F*, with *F* set to zero for negative *F*^2^. The threshold expression of *F*^2^ > σ(*F*^2^) is used only for calculating *R*-factors(gt) *etc*. and is not relevant to the choice of reflections for refinement. *R*-factors based on *F*^2^ are statistically about twice as large as those based on *F*, and *R*- factors based on ALL data will be even larger.

### Fractional atomic coordinates and isotropic or equivalent isotropic displacement parameters (Å^2^)

**Table:**

	*x*	*y*	*z*	*U*_iso_*/*U*_eq_	Occ. (<1)
Cu1	0.22719 (2)	0.09638 (2)	0.73647 (2)	0.02082 (9)	
N1	0.27230 (16)	0.23116 (14)	0.60440 (13)	0.0205 (4)	
N2	0.05462 (16)	0.19388 (14)	0.69210 (13)	0.0193 (4)	
C1	−0.0467 (2)	0.0955 (2)	0.84546 (18)	0.0323 (6)	
H1A	−0.0745	0.0276	0.8442	0.048*	
H1B	−0.0993	0.1359	0.8909	0.048*	
H1C	0.0365	0.0687	0.8683	0.048*	
C2	−0.0512 (2)	0.17993 (18)	0.74111 (18)	0.0257 (5)	
C3	−0.1658 (2)	0.24420 (19)	0.69496 (19)	0.0289 (5)	
H3	−0.2392	0.2375	0.7311	0.035*	
C4	−0.1685 (2)	0.31611 (18)	0.59724 (18)	0.0284 (5)	
H4	−0.2437	0.3563	0.5665	0.034*	
C5	−0.0576 (2)	0.32924 (17)	0.54323 (17)	0.0230 (5)	
C6	−0.0512 (2)	0.40115 (17)	0.44062 (17)	0.0275 (5)	
H6	−0.1233	0.4388	0.4046	0.033*	
C7	0.0580 (2)	0.41503 (18)	0.39547 (18)	0.0273 (5)	
H7	0.0600	0.4603	0.3282	0.033*	
C8	0.1698 (2)	0.36192 (17)	0.44850 (16)	0.0227 (5)	
C9	0.2844 (2)	0.38422 (17)	0.40945 (17)	0.0273 (5)	
H9	0.2910	0.4319	0.3435	0.033*	
C10	0.3853 (2)	0.33573 (18)	0.46848 (17)	0.0268 (5)	
H10	0.4598	0.3539	0.4439	0.032*	
C11	0.3777 (2)	0.25780 (17)	0.56708 (17)	0.0227 (5)	
C12	0.4879 (2)	0.2062 (2)	0.63242 (19)	0.0320 (6)	
H12A	0.4655	0.1590	0.6974	0.048*	
H12B	0.5170	0.2701	0.6412	0.048*	
H12C	0.5518	0.1564	0.6007	0.048*	
C13	0.05311 (19)	0.26752 (16)	0.59555 (16)	0.0203 (5)	
C14	0.16866 (19)	0.28576 (16)	0.54751 (16)	0.0197 (5)	
N3	0.37614 (16)	−0.03179 (14)	0.80310 (14)	0.0224 (4)	
N4	0.23061 (15)	0.15164 (14)	0.86165 (13)	0.0193 (4)	
C15	0.1143 (2)	0.36074 (17)	0.80198 (17)	0.0273 (5)	
H15A	0.1568	0.3556	0.7387	0.041*	
H15B	0.1200	0.4320	0.8166	0.041*	
H15C	0.0292	0.3637	0.7975	0.041*	
C16	0.17194 (19)	0.25310 (17)	0.88480 (16)	0.0209 (5)	
C17	0.1641 (2)	0.25959 (19)	0.98550 (17)	0.0263 (5)	
H17	0.1211	0.3307	1.0002	0.032*	
C18	0.2184 (2)	0.1634 (2)	1.06205 (18)	0.0280 (5)	
H18	0.2099	0.1675	1.1288	0.034*	
C19	0.2881 (2)	0.05700 (18)	1.03802 (17)	0.0237 (5)	
C20	0.3561 (2)	−0.04595 (19)	1.11054 (18)	0.0287 (5)	
H20	0.3505	−0.0479	1.1787	0.034*	
C21	0.4291 (2)	−0.14124 (19)	1.08054 (18)	0.0309 (6)	
H21	0.4723	−0.2079	1.1287	0.037*	
C22	0.4403 (2)	−0.14037 (18)	0.97633 (18)	0.0266 (5)	
C23	0.5219 (2)	−0.23138 (19)	0.9381 (2)	0.0327 (6)	
H23	0.5680	−0.3003	0.9823	0.039*	
C24	0.5322 (2)	−0.21712 (18)	0.8365 (2)	0.0329 (6)	
H24	0.5889	−0.2751	0.8116	0.039*	
C25	0.4591 (2)	−0.11654 (18)	0.76774 (18)	0.0284 (5)	
C26	0.4755 (2)	−0.1021 (2)	0.65667 (19)	0.0356 (6)	
H26A	0.4279	−0.0242	0.6207	0.053*	
H26B	0.4482	−0.1628	0.6403	0.053*	
H26C	0.5611	−0.1099	0.6373	0.053*	
C27	0.29190 (19)	0.05730 (17)	0.93622 (16)	0.0200 (5)	
C28	0.37000 (19)	−0.04304 (16)	0.90432 (17)	0.0211 (5)	
N5	0.19544 (17)	−0.02025 (15)	0.67040 (14)	0.0272 (4)	
C29	0.1858 (2)	−0.08244 (18)	0.62641 (18)	0.0280 (5)	
C30	0.1774 (2)	−0.1635 (2)	0.5691 (2)	0.0381 (6)	
H30A	0.2111	−0.1384	0.5018	0.057*	
H30B	0.0925	−0.1621	0.5656	0.057*	
H30C	0.2230	−0.2435	0.6022	0.057*	
N6	0.1570 (2)	0.8133 (2)	0.92492 (19)	0.0532 (6)	
C31	0.1324 (2)	0.7532 (2)	0.8848 (2)	0.0393 (6)	
C32	0.1015 (3)	0.6748 (2)	0.8345 (2)	0.0493 (7)	
H32A	0.1717	0.6443	0.7951	0.074*	
H32B	0.0336	0.7193	0.7907	0.074*	
H32C	0.0788	0.6091	0.8845	0.074*	
N7	0.1493 (2)	0.4854 (2)	0.0983 (2)	0.0571 (7)	
C33	0.2350 (3)	0.5198 (2)	0.0862 (2)	0.0405 (6)	
C34	0.3440 (3)	0.5640 (3)	0.0680 (3)	0.0680 (10)	
H34A	0.3701	0.5745	−0.0019	0.102*	
H34B	0.4085	0.5073	0.1103	0.102*	
H34C	0.3258	0.6395	0.0835	0.102*	
B1	0.5835 (3)	0.5036 (2)	0.2591 (2)	0.0350 (7)	
F1	0.66138 (15)	0.48303 (17)	0.33530 (13)	0.0687 (5)	
F2	0.46588 (13)	0.56703 (13)	0.28167 (12)	0.0502 (4)	
F3	0.57688 (17)	0.39330 (14)	0.25247 (15)	0.0712 (5)	
F4	0.62598 (14)	0.56564 (15)	0.16773 (13)	0.0653 (5)	
B2	0.1702 (4)	0.0967 (3)	0.3432 (3)	0.0336 (9)	0.825 (2)
F5	0.20526 (15)	0.19999 (12)	0.28948 (11)	0.0480 (4)	0.825 (2)
F6	0.1266 (2)	0.10525 (16)	0.43944 (14)	0.0446 (5)	0.825 (2)
F7	0.07552 (17)	0.08275 (18)	0.29426 (15)	0.0543 (6)	0.825 (2)
F8	0.2690 (2)	−0.00073 (18)	0.34420 (18)	0.0602 (6)	0.825 (2)
B2B	0.2011 (15)	0.0932 (13)	0.3623 (11)	0.0336 (9)	0.175 (2)
F5B	0.20526 (15)	0.19999 (12)	0.28948 (11)	0.0480 (4)	0.175 (2)
F6B	0.2824 (8)	0.0556 (7)	0.4379 (6)	0.0446 (5)	0.175 (2)
F7B	0.0796 (9)	0.1141 (10)	0.4016 (9)	0.0543 (6)	0.175 (2)
F8B	0.2038 (10)	0.0053 (9)	0.3170 (9)	0.0602 (6)	0.175 (2)

### Atomic displacement parameters (Å^2^)

**Table:**

	*U*^11^	*U*^22^	*U*^33^	*U*^12^	*U*^13^	*U*^23^
Cu1	0.02490 (16)	0.01560 (12)	0.02076 (17)	−0.00460 (10)	0.00029 (11)	−0.00420 (9)
N1	0.0239 (10)	0.0171 (8)	0.0204 (10)	−0.0049 (7)	0.0026 (8)	−0.0064 (7)
N2	0.0234 (10)	0.0165 (8)	0.0197 (10)	−0.0072 (7)	0.0024 (8)	−0.0068 (7)
C1	0.0300 (13)	0.0371 (13)	0.0304 (15)	−0.0168 (11)	0.0073 (12)	−0.0053 (10)
C2	0.0280 (13)	0.0222 (10)	0.0316 (14)	−0.0113 (9)	0.0017 (11)	−0.0109 (9)
C3	0.0214 (12)	0.0273 (11)	0.0413 (16)	−0.0100 (9)	0.0011 (11)	−0.0117 (10)
C4	0.0257 (13)	0.0201 (10)	0.0420 (16)	−0.0031 (9)	−0.0104 (11)	−0.0115 (10)
C5	0.0245 (12)	0.0193 (10)	0.0284 (14)	−0.0062 (9)	−0.0035 (10)	−0.0097 (8)
C6	0.0356 (14)	0.0180 (10)	0.0285 (14)	−0.0017 (9)	−0.0153 (11)	−0.0055 (9)
C7	0.0388 (15)	0.0200 (10)	0.0215 (13)	−0.0054 (10)	−0.0049 (11)	−0.0037 (8)
C8	0.0319 (13)	0.0158 (9)	0.0193 (12)	−0.0032 (9)	0.0008 (10)	−0.0067 (8)
C9	0.0395 (14)	0.0189 (10)	0.0197 (13)	−0.0063 (10)	0.0072 (11)	−0.0039 (8)
C10	0.0290 (13)	0.0224 (10)	0.0267 (14)	−0.0088 (9)	0.0092 (11)	−0.0051 (9)
C11	0.0238 (12)	0.0176 (10)	0.0244 (13)	−0.0026 (9)	0.0056 (10)	−0.0070 (8)
C12	0.0243 (13)	0.0325 (12)	0.0345 (15)	−0.0076 (10)	0.0019 (11)	−0.0030 (10)
C13	0.0252 (12)	0.0140 (9)	0.0229 (13)	−0.0050 (8)	0.0007 (10)	−0.0077 (8)
C14	0.0249 (12)	0.0128 (9)	0.0219 (13)	−0.0038 (8)	−0.0002 (10)	−0.0068 (8)
N3	0.0248 (10)	0.0158 (8)	0.0253 (12)	−0.0048 (7)	0.0023 (9)	−0.0052 (7)
N4	0.0187 (9)	0.0168 (8)	0.0216 (11)	−0.0059 (7)	0.0009 (8)	−0.0035 (7)
C15	0.0319 (13)	0.0185 (10)	0.0298 (14)	−0.0018 (9)	−0.0015 (11)	−0.0084 (9)
C16	0.0197 (11)	0.0217 (10)	0.0223 (13)	−0.0074 (8)	0.0030 (10)	−0.0070 (8)
C17	0.0279 (13)	0.0281 (11)	0.0268 (14)	−0.0101 (10)	0.0042 (11)	−0.0127 (9)
C18	0.0308 (13)	0.0395 (13)	0.0195 (14)	−0.0191 (11)	0.0062 (11)	−0.0099 (10)
C19	0.0239 (12)	0.0296 (11)	0.0195 (13)	−0.0161 (9)	0.0013 (10)	−0.0017 (9)
C20	0.0298 (13)	0.0368 (13)	0.0192 (14)	−0.0186 (10)	−0.0047 (11)	0.0036 (9)
C21	0.0290 (13)	0.0283 (12)	0.0311 (16)	−0.0162 (10)	−0.0108 (11)	0.0099 (9)
C22	0.0215 (12)	0.0192 (10)	0.0366 (15)	−0.0101 (9)	−0.0061 (11)	0.0022 (9)
C23	0.0290 (13)	0.0185 (10)	0.0454 (18)	−0.0078 (9)	−0.0078 (12)	0.0028 (10)
C24	0.0242 (13)	0.0177 (10)	0.0544 (19)	−0.0018 (9)	−0.0023 (12)	−0.0092 (10)
C25	0.0262 (13)	0.0215 (10)	0.0361 (16)	−0.0056 (9)	0.0032 (11)	−0.0079 (9)
C26	0.0347 (14)	0.0294 (12)	0.0431 (17)	−0.0020 (11)	0.0056 (13)	−0.0186 (11)
C27	0.0211 (11)	0.0188 (9)	0.0205 (13)	−0.0105 (8)	−0.0007 (10)	−0.0011 (8)
C28	0.0219 (11)	0.0164 (9)	0.0241 (14)	−0.0105 (8)	−0.0005 (10)	0.0010 (8)
N5	0.0371 (11)	0.0189 (9)	0.0232 (11)	−0.0034 (8)	0.0008 (9)	−0.0063 (7)
C29	0.0303 (13)	0.0233 (11)	0.0271 (15)	−0.0045 (9)	−0.0017 (11)	−0.0041 (9)
C30	0.0491 (16)	0.0300 (12)	0.0393 (17)	−0.0109 (11)	−0.0029 (13)	−0.0146 (11)
N6	0.0573 (16)	0.0383 (13)	0.0632 (18)	−0.0169 (12)	0.0008 (13)	−0.0097 (12)
C31	0.0380 (15)	0.0279 (12)	0.0457 (18)	−0.0086 (11)	0.0070 (13)	−0.0037 (11)
C32	0.0546 (19)	0.0414 (15)	0.055 (2)	−0.0162 (14)	0.0111 (16)	−0.0195 (13)
N7	0.0549 (16)	0.0420 (13)	0.078 (2)	−0.0183 (13)	0.0175 (14)	−0.0231 (13)
C33	0.0471 (17)	0.0280 (12)	0.0450 (18)	−0.0105 (12)	0.0058 (14)	−0.0099 (11)
C34	0.053 (2)	0.0423 (16)	0.101 (3)	−0.0180 (15)	−0.0185 (19)	0.0030 (17)
B1	0.0279 (16)	0.0293 (14)	0.043 (2)	−0.0088 (12)	0.0002 (14)	−0.0022 (12)
F1	0.0476 (10)	0.0914 (14)	0.0596 (12)	−0.0102 (10)	−0.0204 (9)	−0.0101 (10)
F2	0.0346 (9)	0.0468 (9)	0.0636 (12)	−0.0047 (7)	0.0037 (8)	−0.0139 (8)
F3	0.0778 (13)	0.0413 (9)	0.0996 (16)	−0.0214 (9)	0.0141 (11)	−0.0272 (9)
F4	0.0410 (10)	0.0648 (11)	0.0601 (12)	−0.0121 (8)	0.0031 (9)	0.0246 (9)
B2	0.042 (3)	0.0327 (15)	0.026 (2)	−0.0028 (16)	0.0009 (17)	−0.0152 (14)
F5	0.0704 (11)	0.0442 (9)	0.0360 (10)	−0.0262 (8)	0.0093 (8)	−0.0143 (7)
F6	0.0712 (14)	0.0388 (9)	0.0236 (11)	−0.0134 (9)	0.0036 (10)	−0.0110 (8)
F7	0.0470 (12)	0.0763 (14)	0.0504 (14)	−0.0257 (10)	−0.0004 (10)	−0.0249 (10)
F8	0.0393 (13)	0.0447 (10)	0.0828 (17)	0.0122 (11)	0.0014 (12)	−0.0196 (10)
B2B	0.042 (3)	0.0327 (15)	0.026 (2)	−0.0028 (16)	0.0009 (17)	−0.0152 (14)
F5B	0.0704 (11)	0.0442 (9)	0.0360 (10)	−0.0262 (8)	0.0093 (8)	−0.0143 (7)
F6B	0.0712 (14)	0.0388 (9)	0.0236 (11)	−0.0134 (9)	0.0036 (10)	−0.0110 (8)
F7B	0.0470 (12)	0.0763 (14)	0.0504 (14)	−0.0257 (10)	−0.0004 (10)	−0.0249 (10)
F8B	0.0393 (13)	0.0447 (10)	0.0828 (17)	0.0122 (11)	0.0014 (12)	−0.0196 (10)

### Geometric parameters (Å, °)

**Table:**

Cu1—N5	2.0123 (18)	C18—C19	1.419 (3)
Cu1—N2	2.0297 (17)	C18—H18	0.9300
Cu1—N3	2.0305 (18)	C19—C27	1.400 (3)
Cu1—N4	2.0348 (17)	C19—C20	1.429 (3)
Cu1—N1	2.1760 (18)	C20—C21	1.362 (3)
N1—C11	1.328 (3)	C20—H20	0.9300
N1—C14	1.374 (3)	C21—C22	1.427 (3)
N2—C2	1.334 (3)	C21—H21	0.9300
N2—C13	1.365 (3)	C22—C28	1.402 (3)
C1—C2	1.496 (3)	C22—C23	1.417 (3)
C1—H1A	0.9600	C23—C24	1.357 (3)
C1—H1B	0.9600	C23—H23	0.9300
C1—H1C	0.9600	C24—C25	1.411 (3)
C2—C3	1.419 (3)	C24—H24	0.9300
C3—C4	1.370 (3)	C25—C26	1.488 (3)
C3—H3	0.9300	C26—H26A	0.9600
C4—C5	1.408 (3)	C26—H26B	0.9600
C4—H4	0.9300	C26—H26C	0.9600
C5—C13	1.415 (3)	C27—C28	1.441 (3)
C5—C6	1.432 (3)	N5—C29	1.128 (3)
C6—C7	1.350 (3)	C29—C30	1.455 (3)
C6—H6	0.9300	C30—H30A	0.9600
C7—C8	1.420 (3)	C30—H30B	0.9600
C7—H7	0.9300	C30—H30C	0.9600
C8—C14	1.405 (3)	N6—C31	1.137 (3)
C8—C9	1.411 (3)	C31—C32	1.458 (4)
C9—C10	1.362 (3)	C32—H32A	0.9600
C9—H9	0.9300	C32—H32B	0.9600
C10—C11	1.417 (3)	C32—H32C	0.9600
C10—H10	0.9300	N7—C33	1.132 (3)
C11—C12	1.494 (3)	C33—C34	1.441 (4)
C12—H12A	0.9600	C34—H34A	0.9600
C12—H12B	0.9600	C34—H34B	0.9600
C12—H12C	0.9600	C34—H34C	0.9600
C13—C14	1.440 (3)	B1—F1	1.367 (3)
N3—C25	1.351 (3)	B1—F4	1.375 (3)
N3—C28	1.359 (3)	B1—F3	1.383 (3)
N4—C16	1.333 (2)	B1—F2	1.390 (3)
N4—C27	1.365 (3)	B2—F8	1.376 (4)
C15—C16	1.495 (3)	B2—F6	1.388 (4)
C15—H15A	0.9600	B2—F5	1.387 (4)
C15—H15B	0.9600	B2—F7	1.397 (4)
C15—H15C	0.9600	B2B—F6B	1.355 (14)
C16—C17	1.407 (3)	B2B—F8B	1.372 (14)
C17—C18	1.365 (3)	B2B—F7B	1.402 (15)
C17—H17	0.9300		
			
N5—Cu1—N2	84.42 (7)	C17—C16—C15	120.15 (18)
N5—Cu1—N3	89.07 (7)	C18—C17—C16	121.2 (2)
N2—Cu1—N3	165.23 (7)	C18—C17—H17	119.4
N5—Cu1—N4	150.90 (7)	C16—C17—H17	119.4
N2—Cu1—N4	98.14 (7)	C17—C18—C19	118.8 (2)
N3—Cu1—N4	81.25 (7)	C17—C18—H18	120.6
N5—Cu1—N1	100.93 (7)	C19—C18—H18	120.6
N2—Cu1—N1	80.17 (7)	C27—C19—C18	116.6 (2)
N3—Cu1—N1	114.15 (7)	C27—C19—C20	119.6 (2)
N4—Cu1—N1	108.09 (6)	C18—C19—C20	123.7 (2)
C11—N1—C14	118.58 (18)	C21—C20—C19	120.5 (2)
C11—N1—Cu1	132.80 (14)	C21—C20—H20	119.8
C14—N1—Cu1	108.16 (13)	C19—C20—H20	119.8
C2—N2—C13	119.90 (19)	C20—C21—C22	121.0 (2)
C2—N2—Cu1	126.65 (15)	C20—C21—H21	119.5
C13—N2—Cu1	112.75 (14)	C22—C21—H21	119.5
C2—C1—H1A	109.5	C28—C22—C23	116.0 (2)
C2—C1—H1B	109.5	C28—C22—C21	119.6 (2)
H1A—C1—H1B	109.5	C23—C22—C21	124.3 (2)
C2—C1—H1C	109.5	C24—C23—C22	119.5 (2)
H1A—C1—H1C	109.5	C24—C23—H23	120.3
H1B—C1—H1C	109.5	C22—C23—H23	120.3
N2—C2—C3	120.5 (2)	C23—C24—C25	121.7 (2)
N2—C2—C1	118.8 (2)	C23—C24—H24	119.1
C3—C2—C1	120.7 (2)	C25—C24—H24	119.1
C4—C3—C2	120.1 (2)	N3—C25—C24	119.7 (2)
C4—C3—H3	120.0	N3—C25—C26	120.1 (2)
C2—C3—H3	120.0	C24—C25—C26	120.2 (2)
C3—C4—C5	120.1 (2)	C25—C26—H26A	109.5
C3—C4—H4	119.9	C25—C26—H26B	109.5
C5—C4—H4	119.9	H26A—C26—H26B	109.5
C4—C5—C13	116.8 (2)	C25—C26—H26C	109.5
C4—C5—C6	124.1 (2)	H26A—C26—H26C	109.5
C13—C5—C6	119.1 (2)	H26B—C26—H26C	109.5
C7—C6—C5	120.9 (2)	N4—C27—C19	123.75 (18)
C7—C6—H6	119.6	N4—C27—C28	116.32 (19)
C5—C6—H6	119.6	C19—C27—C28	119.81 (19)
C6—C7—C8	121.4 (2)	N3—C28—C22	124.35 (19)
C6—C7—H7	119.3	N3—C28—C27	116.23 (18)
C8—C7—H7	119.3	C22—C28—C27	119.3 (2)
C14—C8—C9	116.5 (2)	C29—N5—Cu1	173.4 (2)
C14—C8—C7	119.5 (2)	N5—C29—C30	178.3 (3)
C9—C8—C7	124.0 (2)	C29—C30—H30A	109.5
C10—C9—C8	119.9 (2)	C29—C30—H30B	109.5
C10—C9—H9	120.1	H30A—C30—H30B	109.5
C8—C9—H9	120.1	C29—C30—H30C	109.5
C9—C10—C11	120.5 (2)	H30A—C30—H30C	109.5
C9—C10—H10	119.8	H30B—C30—H30C	109.5
C11—C10—H10	119.8	N6—C31—C32	179.2 (3)
N1—C11—C10	121.0 (2)	C31—C32—H32A	109.5
N1—C11—C12	118.5 (2)	C31—C32—H32B	109.5
C10—C11—C12	120.4 (2)	H32A—C32—H32B	109.5
C11—C12—H12A	109.5	C31—C32—H32C	109.5
C11—C12—H12B	109.5	H32A—C32—H32C	109.5
H12A—C12—H12B	109.5	H32B—C32—H32C	109.5
C11—C12—H12C	109.5	N7—C33—C34	178.5 (3)
H12A—C12—H12C	109.5	C33—C34—H34A	109.5
H12B—C12—H12C	109.5	C33—C34—H34B	109.5
N2—C13—C5	122.4 (2)	H34A—C34—H34B	109.5
N2—C13—C14	118.18 (19)	C33—C34—H34C	109.5
C5—C13—C14	119.38 (19)	H34A—C34—H34C	109.5
N1—C14—C8	123.4 (2)	H34B—C34—H34C	109.5
N1—C14—C13	117.07 (18)	F1—B1—F4	111.3 (2)
C8—C14—C13	119.45 (19)	F1—B1—F3	107.7 (2)
C25—N3—C28	118.60 (19)	F4—B1—F3	109.3 (2)
C25—N3—Cu1	130.46 (16)	F1—B1—F2	110.3 (2)
C28—N3—Cu1	109.46 (13)	F4—B1—F2	109.8 (2)
C16—N4—C27	118.45 (18)	F3—B1—F2	108.5 (2)
C16—N4—Cu1	131.53 (15)	F8—B2—F6	113.5 (3)
C27—N4—Cu1	109.30 (12)	F8—B2—F5	108.5 (3)
C16—C15—H15A	109.5	F6—B2—F5	109.6 (3)
C16—C15—H15B	109.5	F8—B2—F7	106.8 (3)
H15A—C15—H15B	109.5	F6—B2—F7	108.0 (3)
C16—C15—H15C	109.5	F5—B2—F7	110.3 (3)
H15A—C15—H15C	109.5	F6B—B2B—F8B	112.7 (12)
H15B—C15—H15C	109.5	F6B—B2B—F7B	110.6 (12)
N4—C16—C17	120.81 (19)	F8B—B2B—F7B	101.0 (12)
N4—C16—C15	119.04 (19)		

### Hydrogen-bond geometry (Å, °)

**Table:**

*D*—H···*A*	*D*—H	H···*A*	*D*···*A*	*D*—H···*A*
C12—H12C···F8^i^	0.96	2.36	3.120 (3)	135
C18—H18···F5^ii^	0.93	2.36	3.279 (3)	171
C20—H20···F8^ii^	0.93	2.53	3.423 (3)	161
C30—H30A···F8	0.96	2.47	3.375 (4)	158
C30—H30B···F6^iii^	0.96	2.38	3.314 (3)	165
C32—H32B···F7^iv^	0.96	2.37	3.191 (3)	143

Symmetry codes: (i) −*x*+1, −*y*, −*z*+1; (ii) *x*, *y*, *z*+1; (iii) −*x*, −*y*, −*z*+1; (iv) −*x*, −*y*+1, −*z*+1.
